# The Clinical Relevance of Hyperkyphosis: A Narrative Review

**DOI:** 10.3389/fendo.2020.00005

**Published:** 2020-01-24

**Authors:** M. C. Koelé, W. F. Lems, H. C. Willems

**Affiliations:** ^1^Division of Geriatrics, Department of Internal Medicine, Academic Medical Centre Amsterdam, Amsterdam Public Health Research Institute, Amsterdam UMC, Amsterdam, Netherlands; ^2^Department of Rheumatology, Amsterdam Movement Sciences, Amsterdam UMC, Vrije Universiteit Amsterdam, Amsterdam, Netherlands

**Keywords:** hyperkyphosis, kyphosis, older adults, fracture, fall, measurement, review

## Abstract

The kyphosis angle of the thoracic spine tends to increase with aging. Hyperkyphosis is a kyphosis angle, exceeding the normal range. This narrative literature review aims to provide an overview of the current literature concerning kyphosis measurement methods, the etiology and adverse health effects of hyperkyphosis. As of yet, a well-defined threshold for hyperkyphosis is lacking. To attain more generalizability and to be able to compare study results in older adults, we propose to define age-related hyperkyphosis as a Cobb angle of 50° or more in standing position. Hyperkyphosis may be a potentially modifiable risk factor for adverse health outcomes, like fall risk and fractures. Additionally, hyperkyphosis may indicate the presence of osteoporosis, which is treatable. Prospective and intervention studies, using a Cobb angle of 50° as a clear and uniform definition of hyperkyphosis, are warranted to investigate the clinical relevance of hyperkyphosis.

## Introduction

Kyphosis is the curvature of the thoracic spine, formed by the shape of the vertebrae and the intervertebral discs and–in standing position–paraspinal muscle strength. Hyperkyphosis is present when the kyphosis angle exceeds the normal ranges. Apart from the consequences of normal aging, like decreasing muscle strength (1) and degenerative changes of the spine (2), other factors contribute to the increase of the kyphosis angle. Vertebral fractures are present in no less than 40% of the persons with hyperkyphosis (3), and with each vertebral fracture the kyphosis angle increases with 3.8° (4). There is growing evidence showing an association between hyperkyphosis and negative health effects, like a decreased physical performance and a doubled fall risk (4–15).

Currently, numerous kyphosis measurement methods have been used in literature and a clear definition of hyperkyphosis is lacking. If we had a uniform definition of hyperkyphosis, the association with adverse health effects and prognostic value of hyperkyphosis as well as the effectiveness of interventions could be investigated better. This review aims to provide an updated overview of the current studies and to conclude whether hyperkyphosis is relevant for clinical practice. We will discuss the etiology and adverse health effects of hyperkyphosis, and will focus on kyphosis measurement methods. Based on the literature described, we will propose to define hyperkyphosis as a Cobb angle of 50° or more in standing position.

## Methods

We conducted a literature search of PubMed and Embase from 1947 up to now, using the following search terms and derivatives: kyphosis, hyperkyphosis and thoracic spine. We screened the abstracts (9238) and included 74 studies assessing kyphosis measurement methods, the pathogenesis of hyperkyphosis or the association with clinically relevant outcomes. We excluded non-English studies, duplicate or overlapping articles intervention studies assessing the effect of surgical procedures and studies in children or in participants with hyperkyphosis caused by disease and scoliosis.

### Kyphosis Measurement Methods

**Figure 1:**
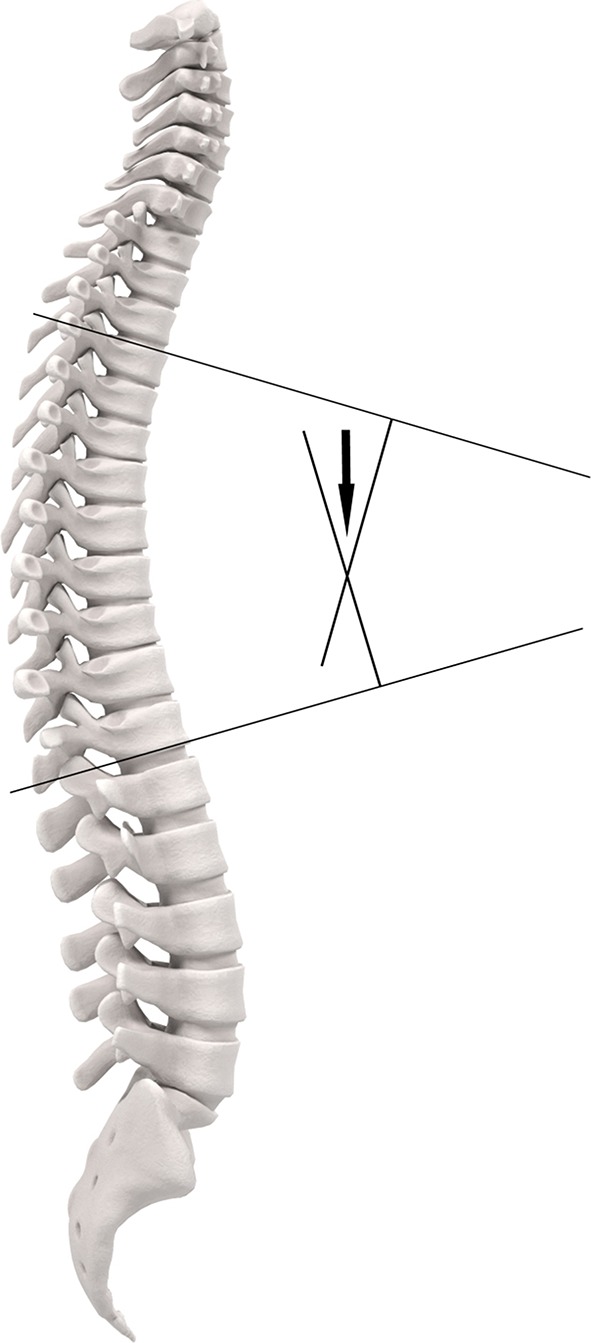
Cobb angle.

The Cobb angle is considered to be the current gold standard method to measure kyphosis (16). Initially, the Cobb angle was developed to assess scoliosis angles. By modifying the direction of radiographic imaging from frontal to sagittal projection, the Cobb angle became useful to assess kyphosis angles (17). The vertebrae superior to the fourth thoracic vertebra (T4) are often less well visible due to over projection of other structures. Therefore, commonly the angle between T4 and T12 is used. The Cobb angle is measured by drawing a line through the superior endplate of T4 and a second line through the inferior endplate of T12. At the intersection of these two lines, the Cobb angle can be measured (Figure 1).

**Figure 2:**
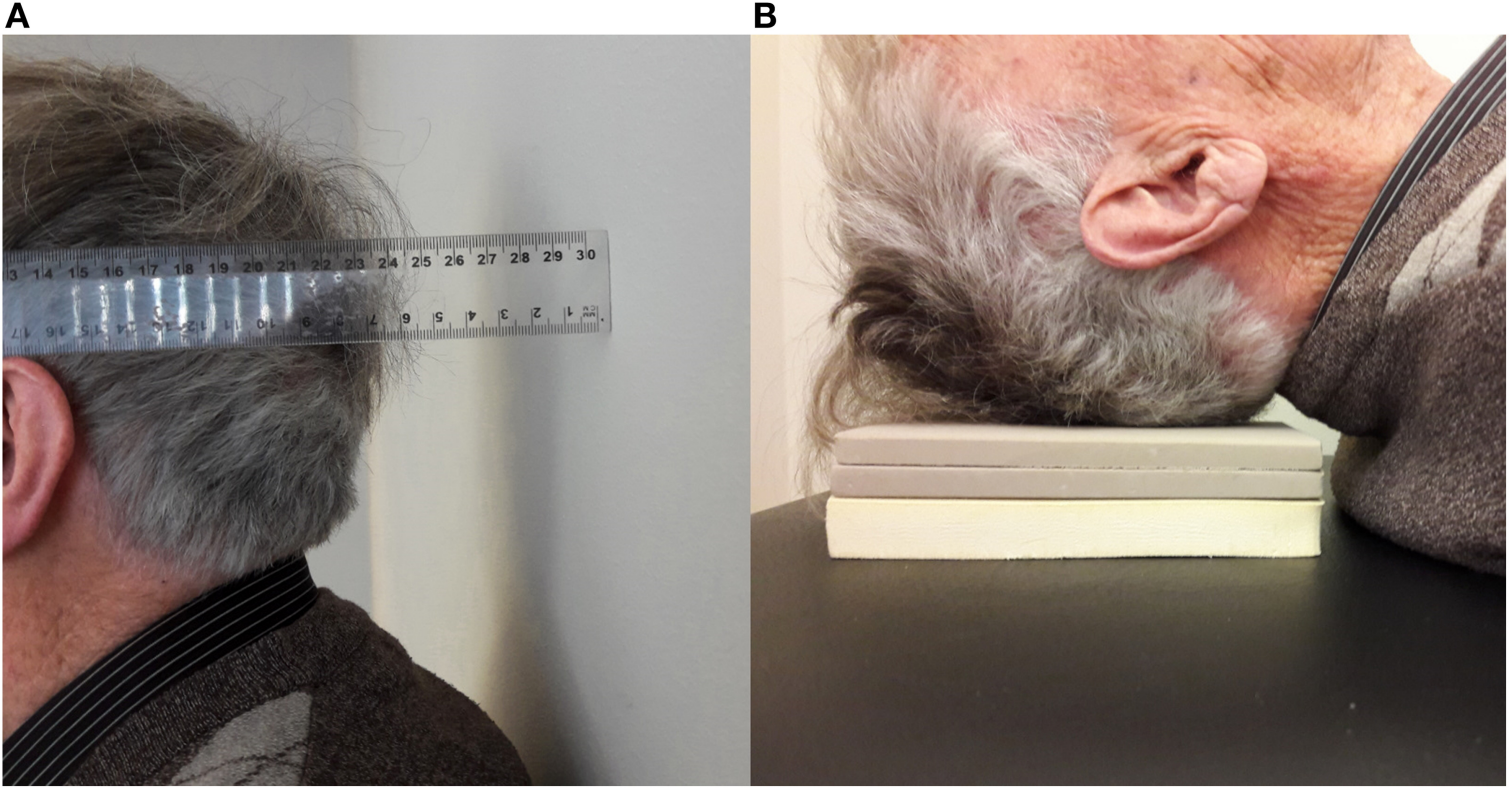
**(A)** Occiput-to-wall distance and **(B)** Blocks method.

In addition to the Cobb angle, several clinimetric kyphosis measurement methods have been developed. A protractor is used to measure the kyphosis angle with the Debrunner kyphometer (18), goniometer (19), arcometer (20), and inclinometer (21). The upper arm of the protractor is placed on C7 or T1, and the lower arm on T12. Two other devices—the flexicurve ruler (22) and the spinal mouse (23)—document the contour of the spine. The flexicurve ruler is molded to the spine from C7 in caudal direction. The kyphosis index is the width divided by the length of the thoracic curve. The spinal mouse is a device with accelerometers, detecting distance and changes of inclination while rolled over the spine. Finally, the occiput-to-wall distance (OWD) and the blocks method are used to quantify kyphosis (Figure 2).

Every measurement method has its own characteristics, advantages and disadvantages. While the Cobb angle has the advantage of providing information on the anatomy of the vertebrae and spinal alignment, radiation exposure is inevitable. High interrater and intrarater reliability have been described in studies with well-trained examiners to score the Cobb angles. The correlation coefficients range from 0.80 to 1.00 (4, 7, 24, 25), which may be expected to be lower when performed in clinical practice by less experienced examiners. The clinimetric measurement methods make radiation exposure redundant. Some clinimetric measurement methods are easy to use in clinical practice and the result instantly available.

However, the correlation with the Cobb angle ranges extensively from low (0.28) to high (up to 0.92) (7, 19, 24, 26–28). These large differences may be explained by the variety in kyphosis measurement methods regarding the position of the person during measurement and which part of the spine is measured. In supine position, the back is passively stretched and the influence of muscle strength may be diminished when compared to a standing position. Most of the measurement methods only measure the curve of the thoracic spine. Yet the blocks method also takes the cervical spine into account, and the OWD is influenced by the posture of the patient when standing.

Thus, in addition to the gold standard kyphosis measurement–the Cobb angle–various kyphosis measurement methods have been used in literature. Correlation among the measurement methods ranges extensively, possibly reflecting the large differences between methods.

### Definition

With aging, kyphosis tends to increase. In younger adults, the Cobb angle averages from 20 to 29° (17). After the fourth decade, the kyphosis angle increases (17). In two cohort studies among older women, the Cobb angle increased with 2.6° in 3 years and 7° in 15 years (5), and 3.9 in 4 years (29). The mean kyphosis angle ranges from 35 to 38° in adults, aged 65 years and older (30, 31). In a cohort of older End Stage Renal Disease patients, the mean Cobb angle was 41° (32). In another cohort with women aged 65 years and older, the mean kyphosis angle increased with age from 47 to 52° (33). However, these values were measured in a cohort with underlying osteoporosis, with potentially more vertebral fractures and thereby a higher mean Cobb angle than the general population.

Currently, a well-defined threshold, differentiating between normal kyphosis and hyperkyphosis, is lacking. In some studies, the 95th percentile of the Cobb angle in younger adults is used as threshold (17). As the mean angle in older adults ranges from 35 to 52°, the prevalence of hyperkyphosis may be overestimated in these studies. In other studies, a higher threshold value of 50° is used (6, 14, 29, 34). When using these threshold values, hyperkyphosis is present in 20–40% of the older adults (7, 27, 35, 36). Even higher prevalences of up to 55% are reported in a geriatric population (14). The abundance of kyphosis measurement methods makes a large number of other definitions of hyperkyphosis necessary. Yet for some measurement methods no threshold value could be found in literature, and for some kyphosis measurement methods the threshold value differs between studies. McDaniels-Davidson et al. defined hyperkyphosis as 54°, measured with the Debrunner kyphometer and 17, measured with the Flexicurve ruler (6). Hyperkyphosis, measured with the OWD, is defined in literature as 4 cm (15, 37) or 5 cm (14, 38). Different threshold values to define hyperkyphosis are used for the blocks method: ≥1 block (39), ≥2 blocks (36, 40), and ≥4 blocks (6).

In conclusion, a large number of kyphosis measurement methods with various threshold values for hyperkyphosis are used in addition to the gold standard method, the Cobb angle Generalizability and comparison of study results is therefore limited. Though preferable in order to pursue more uniformity in hyperkyphosis research, differentiating between normal and abnormal kyphosis angles remains difficult.

## Pathogenesis

### Vertebral Fractures and Degenerative Disc Disease

The pathogenesis of hyperkyphosis has not yet been completely elucidated. Anterior wedging of the vertebrae and asymmetrical compression of the intervertebral discs may result in an increase of the kyphosis angle (41). In adults with vertebral fractures, hyperkyphosis is more prevalent (3, 33, 42). Kado et al. showed that with each compression fracture, the kyphosis angle increased with 3.8° (4). Yet, only 40% of the patients with hyperkyphosis has vertebral fractures (3), which suggests that other risk factors may play a role. With aging, the intervertebral discs desiccate. This process is referred to as degenerative disc disease. Manns et al. showed a significant correlation between anterior disc height and kyphosis angle (*r* = −0.34, *p* < 0.001) and a negative correlation to age (*r* = −0.30, *p* = 0.01), potentially indicating that disc degeneration is not a disease, but merely part of normal aging (43).

As most studies are cross-sectional, it is unclear whether degenerative disc disease is a cause or consequence of hyperkyphosis. Only Kado et al. has reported an association between degenerative disc disease and hyperkyphosis–and not kyphosis progression–in a longitudinal study. However, due to the retrospective design, no conclusions could be drawn on causality of the two phenomena (4). Another possibility is that–rather than being cause or consequence–degenerative disc disease and hyperkyphosis enhance each other. Anterior compression of the intervertebral discs may increase the kyphosis angle, and this in turn may enhance further compression of the discs.

### Muscle Strength

Besides the vertebrae and intervertebral discs, paraspinal muscle strength may influence kyphosis. Back extensor muscle strength has been shown to be inversely correlated to kyphosis (44, 45). Hyperkyphosis may be an indicator of frailty, as grip strength is one of the Fried criteria. However, the association between kyphosis angle and grip strength remains controversial, as some cohort studies report a positive association (9, 40), and others a negative association (5, 46).

### Genetic Predisposition

In some heritable diseases like Scheuermann's disease, hyperkyphosis is seen at an early age. Kado et al. reported that independent of vertebral fractures and bone mineral density (BMD), women with 1–2 parents with hyperkyphosis had on average 2.6° worse kyphosis angle compared to women with parents without hyperkyphosis (4). A twin study among 241 twins found a heritability estimate of 61% (95%CI 46–72) (47). In the Framingham study, the heritability estimate was reported to be 54% (95%CI 43–64%) (48). Mouse knock-out and transgenic models show that hyperkyphosis may be enhanced by mutations in the genes involved in DNA repair and delaying senescence (49, 50).

## Adverse Health Effects of Hyperkyphosis

### Physical Performance

A large number of cohort studies has investigated the association of the kyphosis angle and physical performance (4, 5, 7–11, 51). In all studies, except the study of Demarteau et al., multivariate analyses were performed to adjust for age and comorbidity including vertebral fractures or BMD. Only Katzman et al. investigated this association prospectively in a large cohort of women (mean age 68 years) (5). Performance time on the Timed Up and Go test (TUG) increased with increasing kyphosis angle. Although statistically significant, the effect size of this difference is very small. Similar to this study, all studies consistently report a statistical significant lower physical performance in hyperkyphotic participants, potentially indicating publication bias. Yet, various kyphosis measurement methods and physical performance tests have been used, and reported differences are small. Therefore, the clinical relevance of this association between hyperkyphosis and physical performance is questionable.

### Falls

The majority of studies, including two studies with a prospective design, show that hyperkyphosis is associated with falls (6, 12, 14, 15, 39). One relatively small study (*n* = 73) may have overestimated the association, as age was not added in the multivariate analyses (12). One prospective and two cross-sectional studies found no association between hyperkyphosis and falls (13, 32, 52).

The underlying cause of the increased fall risk in older adults with an increased kyphosis angle may be balance disruption due to a forward shift of the center of gravity of the body (53). Indeed, older adults with hyperkyphosis have an increased postural sway, wider stance and reduced gait speed (38, 53). However, conflicting results have been reported on the association between balance and hyperkyphosis. Some studies report a positive correlation (13, 34, 54), while others found no correlation between hyperkyphosis and impaired balance (7, 55). This difference may partly be explained by the balance test used, as clinical tests like used in one of the two negative studies (7), may be less sensitive to detect balance problems than post-urography, which is used in the studies reporting a positive association. Significant methodological limitations of the before mentioned studies may be a second reason for the conflicting results of the studies (7, 34, 54, 55). Only the study of Ishikawa et al. adjusted for potential confounders (13).

In conclusion, the majority of studies shows that in adjusted analyses, hyperkyphosis is associated to falls. Whether impaired balance is the underlying mechanism of the increased fall risk in persons with hyperkyphosis, is currently unknown.

### Fractures

Hyperkyphosis increases pressure on the anterior part of the vertebrae. Consequent vertebral fractures may therefore be expected. Huang et al. indeed reported an increased risk of vertebral fractures in women (mean age 71 years) with hyperkyphosis (adjusted OR 1.7, 95%CI 1.0–3.0) (25). This is confirmed in another longitudinal study of Kado et al. in women (mean age 69 years) with hyperkyphosis (HR 1.50, 95%CI 1.10–2.06, model adjusted for age and BMD). Change of the Cobb angle was also independently associated to fracture risk (HR 1.28, 95%CI 1.06–1.55) (29). Opposite to these results, one large cohort study among older women (mean age 68 years) with low BMD or prevalent vertebral fracture reported no association (IRR 1.08, 95%CI 0.96–1.22, model adjusted for age and BMD). Change of the kyphosis angle was not associated with fracture risk (42).

Thus, conflicting results regarding the association between hyperkyphosis and future fractures have been reported in women with low BMD (25, 29, 42). These differences may be explained by the difference in regression models applied in the studies. The studies reporting a positive association have applied logistic regression and Cox regression, while the study reporting no association used Poisson regression. The chance of a future vertebral fracture is dependent on previous fractures. Therefore, the standard error is smaller and the confidence interval too narrow, which makes the test statistic too high and the estimated effect of the predictor on the outcome too high. Poisson regression corrects for the type I error caused by the correlation between a first fracture and next fractures. Therefore, the studies of Huang et al. and Kado et al. may have overestimated the effect of hyperkyphosis on fracture incidence.

### Pulmonary Function

Literature on the association of hyperkyphosis with pulmonary function is scarce. Increased thoracic kyphosis may cause mechanical restriction of pulmonary function, as reported in all four articles included in the systematic review of Harrison et al. (56). Older adults with hyperkyphosis have more often dyspnoea and decreased vital capacity (57, 58) and forced expiratory volume (58, 59). Lombardi et al. was the only study, in which correlations were unadjusted (58). The retrospective study of Lee et al., found no association with acute respiratory failure in 51 hyperkophotic participants (unadjusted HR 3.20, 95%CI 0.86–12.14) (60).

Thus, consistent results on the association between hyperkyphosis and pulmonary function have been reported, though internal and external validity of the studies is limited. Whether hyperkyphosis leads to a higher incidence of diseases like pneumonia or COPD, is yet unknown.

### Mortality

Four large cohort studies report that hyperkyphosis is associated with a higher all-cause mortality (32, 36, 61, 62). In the Rancho-Bernardo cohort, the odds ratio was 1.40 (95%CI 1.07–1.82) in the multivariable model adjusted for age, gender, smoking, physical activity and BMD (36). Goto et al. reported an association between hyperkyphosis and mortality in end stage renal disease patients, yet they may have overestimated the association as they did not adjust for potential confounders (32). Mortality rates increase with increasing kyphosis angle in older women with osteoporosis in the for age and comorbidity adjusted model (61), possibly reflecting the number of osteoporotic fractures and thus the severity of osteoporosis.

### Pain

Remarkably, only in a few studies the association between hyperkyphosis and pain has been investigated (33, 63–65). Three out of four studies adjusted for age (33, 63, 64). All studies except Ettinger et al. (63) report a positive correlation or association with pain.

### Quality of Life

As mentioned above, several negative health conditions, like pain and lower physical performance, have been linked to hyperkyphosis. Lower quality of life may therefore be a logical consequence. Less satisfaction with life in participants with a larger kyphosis angle has been described (66–68). However, results are difficult to interpret due to significant methodological limitations. Only Martin et al. adjusted for potential confounders, i.e., age and BMD (66).

## Discussion and Conclusion

Hyperkyphosis is common in older adults. This review reveals several shortcomings in the literature concerning the clinical relevance of hyperkyphosis.

First of all, a well-defined threshold for hyperkyphosis is lacking. Yet, in order to attain more uniformity in research, applying one clear definition of hyperkyphosis is essential. As the Cobb angle is the gold standard kyphosis measurement method, a definition of hyperkyphosis based on the Cobb is preferable. The mean kyphosis angle has been reported to range from 35 to 42° in adults aged 65 years and older (30–32, 69), with a larger mean angle of 47–52° in older women with osteoporosis. We need to take the measurement error into account, as the interrater and intrarater variability ranges from 3 to 5° (70, 71). Defining hyperkyphosis based on means and interrater and intrarater variability may be preferable, as a definition based on the association with adverse health outcomes would only be applicable in similar populations. Based on the range of the mean kyphosis angle in older adults and interrater and intrarater variability, we propose to define hyperkyphosis as a Cobb angle of 50° or more in standing position. Additionally, identifying a pre-stage of hyperkyphosis–a Cobb angle ranging from 40 to 50°–may facilitate early recognition and potential intervention.

Secondly, many cohort studies report an association between hyperkyphosis and adverse health effects. However, most studies have a cross-sectional design and some outcome measures have been scantly investigated. Moreover, most studies have been performed in a population with osteoporosis. In order to gain knowledge on the consequences of hyperkyphosis, more prospective studies are warranted in other populations. While literature concerning the consequences of hyperkyphosis may be limited, osteoporotic vertebral fractures have consistently been identified as one of the causes of hyperkyphosis. Therefore, hyperkyphosis may be a clear clinical sign of the presence of osteoporosis. As osteoporosis is treatable, early recognition is highly important to prevent future fractures and the accompanying health-related problems. Finally, a few small intervention studies have shown that hyperkyphosis in itself is treatable through targeted training of back extensor muscles or yoga (72–75).

In conclusion, hyperkyphosis is a clinical sign of the presence of osteoporosis, and a potentially modifiable risk factor for adverse health outcomes. Prospective and intervention studies, using a Cobb angle of 50° as a clear and uniform definition of hyperkyphosis, are warranted to investigate the clinical relevance of hyperkyphosis.

## Author Contributions

MK performed the literature search and wrote the draft. MK and HW read the articles. WL and HW reviewed the draft of the article and provided expertise for revisions. All authors approved the submitted version of the manuscript.

### Conflict of Interest

The authors declare that the research was conducted in the absence of any commercial or financial relationships that could be construed as a potential conflict of interest. The reviewer JN declared a shared affiliation, with no collaboration, with one of the authors, WL, to the handling editor at the time of the review.
